# Adolescent Social Media Use and Depression: A Person-Centered Approach

**DOI:** 10.1007/s10578-025-01819-1

**Published:** 2025-03-08

**Authors:** Shanyan Lin, Claudio Longobardi

**Affiliations:** Dipartimento di Psicologia, Università degli Studi di Torino, Turin, Italy

**Keywords:** Specific social media use, Self-esteem, Self-concept clarity, Depression, Adolescents, Person-centered approach

## Abstract

Many previous studies have investigated the effects of social media use (SMU), including both general SMU and specific types of SMU (e.g., active and passive SMU), on adolescents’ depression. However, very few of these studies have employed a person-centered approach. To address this gap, this study conducted a latent profile analysis using five SMU indicators (i.e., problematic SMU, SMU intensity, active SMU, passive SMU, and nighttime SMU) to identify potential SMU patterns among adolescents. The participants were 986 Italian students (525 girls, 53.2%) from senior high schools, aged 13 to 20 years old (*M* = 16.84, *SD* = 1.60). Five SMU profiles emerged: (1) the *Active users* (*n* = 126, 12.8%), (2) the *Low-intensity passive users* (*n* = 97, 9.8%), (3) the *Passive users* (*n* = 251, 25.5%), (4) the *Problematic active users at night* (*n* = 358, 36.3%), and (5) the *Highly problematic active users at night* (*n* = 154, 15.6%). The relative mediating effects of adolescents’ self-esteem and self-concept clarity were found in the relationships between SMU profiles and depression. This study highlights the heterogeneity of SMU patterns among adolescents, their association with depressive symptoms, and the potential underlying mediating mechanisms.

## Introduction

During the last two decades, the pervasive social media use (SMU) has become almost indispensable in the daily lives of people across many countries. This prevalence is particularly pronounced among adolescents, with the majority of them regularly engaging with platforms such as TikTok, Instagram, WhatsApp and so on [1, 2]. Many previous reviews and meta-analyses have emphasized the associations between adolescent SMU and various facets of mental health, such as depressive symptoms [3], well-being and ill-being [4], psychiatric disorders [5], and so on (for an overview of reviews/meta-analyses, see [6, 7]). However, many of the prior empirical studies employed a variable-centered approach, potentially overlooking individual differences in SMU patterns. Consequently, the application of methods that take individual differences in SMU patterns into account, such as a person-centered approach, might provide valuable insights. This approach could enable a deeper exploration into the impact of SMU on adolescents’ developments and adjustments, as well as providing a clearer understanding of the mechanisms underlying these relationships.

### Social Media Use and Depression Among Adolescents

A substantial body of evidence has explored the relationship between general SMU and depressive symptoms (for a review or meta-analysis, see [8, 9]). The significant association is supported by a large number of cross-sectional studies (e.g., [10–12]), several rigorous longitudinal studies (e.g. [13, 14],), and a handful of well-designed experimental studies (e.g., [15, 16]). Specifically, in a three-wave longitudinal study by Tandoc and Goh [14], increased Facebook use at the initial time point was associated with increased depression levels at the third time point. Hunt et al. [16] observed that limiting daily social media use to approximately 30 min over a three-week period led to a significant reduction in depression levels in the experimental group compared to the control group. However, meta-analyses (e.g., [17]) have also shown that the effect size in the relationship between SMU and depressive symptoms is modest, coupled with considerable heterogeneity. Therefore, the relationship between SMU and depression remains inconclusive. This highlighted the necessity to further investigate the relationship between specific types of SMU and depressive symptoms since most previous studies only focused on general SMU.

When examining the association between specific types of SMU and depression, several studies have investigated this relationship. First, in contrast to general SMU intensity or frequency, problematic SMU or social media addiction (SMA)—characterized by problematic or addictive ways of SMU—is relatively consistently linked to more depressive symptoms [15, 18–20]. Second, according to the active–passive model of SMU [21], SMU can be categorized into two specific types of use, including active SMU and passive SMU. Active SMU refers to activities on social media that facilitate information exchange (e.g., targeted private messages, non-targeted posts), while passive SMU is a type of passive consumption of the content on social media. Active SMU was associated with fewer depressive symptoms, whereas passive SMU was associated with more depressive symptoms [10, 12, 22, 23]. Finally, SMU at certain times (e.g., at night) has been found to be particularly detrimental to sleep quality [24–26] which in turn predicted more depression or a combination of depression and anxiety problems (for a review, see [27]).

Although the relationship between the specific forms/periods of SMU and its effects is relatively clearer and robust, conflicting results can also be found in the literature. For example, although many previous studies found a relationship between active/passive SMU and well-/ill-being (e.g., [12, 28, 29]), this relationship was not supported in many other studies (for a critical scoping review, see [6, 7]). Focusing on only one singular type of specific SMU might still be too coarse to capture the nuanced relationships present. Therefore, in the present study, we adopted a comprehensive approach, in which we examined multiple facets of SMU simultaneously—including its intensity, problematic/addictive use, active versus passive engagement, and nighttime use—to delineate potential patterns of SMU. We then examined the associations between these identified patterns and depressive symptoms among adolescents, and also exploring the potential mediating mechanisms.

### The Mediating Role of Self-Esteem and Self-Concept Clarity

The extent of depression in adolescents with different SMU patterns may vary, and their self-concept may serve as a mediating factor in this relationship. Self-esteem—as the content aspect of self-concept—is defined as a “favorable or unfavorable attitude towards the self” [30], while self-concept clarity—as the structural aspect of self-concept—is defined as “the extent to which self-beliefs are clearly and confidently defined, internally consistent, and stable” [31]. In the first stage of the proposed mediating process, SMU appears to be related to adolescents’ self-esteem and self-concept clarity. Interpersonal communication and the social environment are pivotal for identity development which is a key task during adolescence [32]. In addition to the traditional offline communication and environment, social media offer young people an additional cyberspace in which they can explore their self-concept. Through online platforms, they can easily cultivate and maintain social connections, express themselves through various means (e.g., photos, texts, and videos), receive feedback from peers and individuals with diverse backgrounds and perspectives, and potentially be influenced by content they encountered within their digital networks. This can have a detrimental effect on self-concept development and disintegrate their personality according to the self-concept fragmentation hypothesis [33, 34]. Empirical evidence also supports the relationship between SMU and users’ self-concept. Many previous studies have shown that self-esteem and self-concept clarity are influenced by the specific types/periods of SMU, including problematic SMU or SMA [35, 36], SMU intensity [37, 38], active/passive SMU [28, 29, 38, 40], and nighttime SMU [25].

In the second stage of the proposed mediating process, adolescents’ self-esteem and self-concept clarity may further influence their depressive symptoms. According to Beck’s cognitive theory of depression [41, 42], maladaptive self-schemata and self-cognition constitute the cognitive vulnerability to depression. Adolescents with higher levels of self-esteem and/or a clearer self-concept may have fewer depressive symptoms or a lower likelihood of developing a depressive disorder. Consistently, a large body of empirical evidence also suggests that self-esteem and self-concept clarity negatively predict or influence depression among adolescents [43–49]. For example, cross-lagged regression analyses on four repeated assessments revealed that self-esteem negatively predicted later depression level in adolescence and young adulthood [46]. A meta-analysis of longitudinal studies also found that the effect of self-esteem on depression was significantly stronger than the effect of depression on self-esteem [47]. In addition, self-concept clarity was found negatively associated with depression among adolescents [45] and late adolescents [44]. Based on the theoretical and empirical evidence for the first and second stages of the mediating process, it is hypothesized that self-esteem and self-concept clarity play the mediating role in the relationship between SMU and depression.

### The Person-Centered Perspective

The relationship between SMU and its effects on adolescents has been investigated in many previous studies adopting variable-centered approach. This approach assumes that each individual in the sample can be represented by an equal set of averaged parameters [50]. However, the variable-centered approach ignores the fact that adolescents may have different patterns of social media use when considering multiple indicators of SMU, such as problematic SMU, SMU intensity, active/passive SMU, and nighttime SMU. Compared to the variable-centered approach, the person-centered approach assumes that the observed parameters or relationships are not always the same for each individual since there may exist potential subgroups with different sets of parameters in the sample [50–52]. Through the person-centered approach, it becomes feasible to identify the adolescents that exhibiting similar SMU patterns. This facilitates a more holistic and comprehensive exploration of the associations between these patterns and developmental outcomes in adolescents. Although some previous studies (e.g., [53, 54]) have utilized person-centered methods to investigate adolescent media use patterns, these studies primarily concentrated on general behaviors like smartphone use [53] and screen-based sedentary activities [54]. Research specifically targeting different types of specific SMU is scarce. Therefore, in the present study, the potential patterns of adolescent specific SMU behaviors, as well as how they are related to depression, were explored using one of the person-centered approaches (i.e., latent profile analysis).

### The Present Study

Although the relationship between SMU and its effects on adolescents psychosocial development has been investigated in many previous studies, most of them adopted variable-centered approach focusing on one or two specific aspects of SMU. Potential subgroups with similar patterns of social media use among adolescents, as well as the heterogeneity in their relationships with depressive symptoms, have hardly been explored. To fill these gaps, the current study was conducted based on the theretical and empirical evidence mentioned aboved. The aims of the current study were: (1) to indentify the patterns of SMU among adolescents using the indicators of problematic SMU, SMU intensity, active SMU, passive SMU, and nighttime SMU; (2) to explore the relationships between the potential SMU profiles and depression; and (3) to examine the mediating roles of self-esteem and self-concept clarity.

## Method

### Participants and Procedure

The participants were 986 Italian adolescents from senior high schools. They were enrolled in five grades from Grade 9 to Grade 13. There were 525 female students (53.2%) in the sample. The average age of the participants was 16.84 years (*SD* = 1.60), ranging from 13 to 20 years. Further details of participant demographics can be found in Table 1. Prior to the data collection, ethical approval was obtained from the ethics committee at the authors’ affiliated university. Participants were recruited through convenience sampling, with schools being contacted via public contact information (e.g., email) or through the authors' personal networks. Eight public schools located in northern Italy agreed to participate in this study. In the spring and early summer of 2023, Trained research assistants who were master students of psychology visited these high schools to collect data. Participants completed the questionnaire in their classrooms. The rights of the participants were fully protected. The questionnaire was administered anonymously and the collected data were exclusively used for research purposes. Participation in this study was completely voluntary and carried no incentives or rewards.

**Table 1:** Students’ demographic information

	Frenquency	Percentage
Gender		
Female	525	53.2%
Male	461	46.8%
Age		
13	1	0.1%
14	95	9.6%
15	138	14.0%
16	181	18.4%
17	146	14.8%
18	277	28.1%
19	131	13.3%
20	17	1.7%
Grade^A^		
Grade 9	147	14.9%
Grade 10	164	16.6%
Grade 11	196	19.9%
Grade 12	126	12.8%
Grade 13	353	35.8%
Highest education level of parents^B^		
Middle school and below	131/197	13.3%/20.0%
Vocational school	195/292	19.8%/29.6%
High school	340/251	34.5%/25.5%
Bachelor and above	315/240	31.9%/24.3%
Missing	5/6	0.5%/0.6%

^A^There are five grades in a typical Italian high school from grade 9 to grade 13. ^B^ Highest education level of parents: on the left of the slash line are maternal highest education level information and on the right of the slash are paternal information

### Measures

#### Social Media Use

##### Problematic Social Media Use

The Bergen Social Media Addiction Scale (BSMAS, [55]), revised by Monacis et al. [56], was used to measure the level of problematic social media use. The BSMAS is a 5-point scale (1 = *Very rarely*, 5 = *Very often*) with 6 items (e.g., “How often during the last year have you felt an urge to use social media more and more?”). The sum of ratings on all items was taken as the final score. In the current sample, the Cronbach’s alpha was 0.72.

##### Social Media Use Intensity

The intensity of social media use was measured with the Social Media Use Intensity Scale (SMUIS, [57]) which is a 7-point scale (e.g., 1 = *Never or less than once*, 7 = *More than 40 times*) with 4 items (e.g., “How many times a day do you check social network sites?”). The final score of SMUIS was the average of the 4 items. In the current sample, the Cronbach’s alpha was 0.72.

##### Active Social Media Use

The active social media use was measured using a 7-point scale (1 = *Never*, 7 = *Several times per day*) with five items (e.g., “How often do you send someone a personal message on social media?”). This scale was adapted from the Multidimensional Scale of Facebook Use [58]. The average of all the items rating were regarded as the final score. The Cronbach’s alpha in the current sample was 0.79.

##### Passive Social Media Use

The passive social network site use question [59] was used. This is a 5-point scale (1 = *Strongly disagree*, 5 = *Strongly agree*) with five items (e.g., “I often browse social network sites but don’t post status updates”). The sum of all the items was calculated as the final score. The Cronbach’s alpha was 0.63.

##### Nighttime Social Media Use

Social media use at nighttime was measured using the Night-time Specific Social Media Use Scale (NSSMUS, [25]) with seven items (e.g., “In the past month, how often have you used social media in bed?”). Ratings on all items were summed to produce the final score ranging from 0 to 31. The Cronbach’s alpha of the NSSMUS in the current sample was 0.73.

#### Self-Esteem

The Rosenberg Self-esteem Scale (RSES), developed by Rosenberg [30], was adopted to measure self-estem. Participants were asked to rate on a 4-point scale, ranging from 1 (*Strongly disagree*) to 4 (*Strongly agree*). The RSES comprises 10 items (e.g., “I feel that I am a person of worth, at least on an equal plane with others”). The sum of ratings on all items was calculated as the final self-esteem score, with a higher value indicating a higher level of self-esteem. In the present sample, the internal consistency of the RSES was sufficient (the Cronbach’s alpha = 0.87).

#### Self-Concept Clarity

The self-concept clarity was measured by the Self-Concept Clarity Scale (SCCS). The SCCS was developed by Campbell et al. [31]. The SCCS contains 12 items (e.g., “On one day I might have one opinion of myself and on another day I might have a different opinion”, reverse-coded) which were rated on a 5-point scale (1 = *Strongly disagree* to 5 = *Strongly agree*). The final level of self-concept clarity was calculated as the sum of all the item ratings, with a higher value representing better self-concept clarity. In the current sample, the internal consistency of SCCS was sufficient (the Cronbach’s alpha = 0.86).

#### Depression

The depression was measured by the depression subscale of the Depression Anxiety and Stress Scales (DASS-21) [60],revised by [61]. This subscale of the DASS-21 consists of 7 items. Students had to rate them on a 4-point scale (0 = *Never* to 3 = *Always*). An example of the items is that “I felt that I had nothing to look forward to”. The sum of all the ratings on 7 items was taken as the final score, with a higher value representing higher depression level. In the present sample, the Cronbach’s alpha of the depression subscale was 0.85, which indicates sufficient consistency.

### Data Analysis

The data were analysed in SPSS 29 and Mplus 8.3 [62]. First, descriptive and correlative analysis were conducted, and the direct effect of specific types/periods of social media use on adolescents’ depression was simultaneously examined to preliminarily explore the relationships between the variables. Second, the latent profile analysis was performed to identify the social media use profiles among adolescents. The indicators were problematic social media use, social media use intensity, active social media use, passive social media use, and nighttime social media use. Profile numbers ranging from one to six were analysed. The final number of profiles was selected based on multiple indices, including the Akaike Information Criterion (AIC), the Bayesian Information Criterion (BIC), the adjusted Bayesian Information Criterion (aBIC), entropy (≥ 0.7, [63]), the Lo-Mendell-Rubin adjusted likelihood ratio test, the bootstrapped likelihood ratio test (BLRT), and the smallest profile size (≥ 5% of the total sample) [64]. Then, multivariate analysis of variance (MANOVA) was performed to analyse the indicator differences between the profiles [65, 66]. Finally, the mediating role of self-esteem and self-concept clarity in the relationship between the identified social media profiles and adolescent depression was further explored by performing the mediation analysis with a multicategorical antecedent. The 95% confidence interval (CI) for the relative indirect effect was generated using a bootstrap sample of 5000. A 95% CI that excludes zero indicates a significant relative indirect effect.

## Results

### The Preliminary Results

The direct effects of the five specific types/periods of SMU on adolescent depression were analysed simultaneously. The path analysis with multiple independent variables revealed that problematic SMU (β = 0.30, SE = 0.04, 95% CI = [0.22, 0.37], *p* < 0.001), passive SMU (β = 0.12, SE = 0.04, 95% CI = [0.04, 0.19], *p* = 0.001), and nighttime SMU (β = 0.16, SE = 0.04, 95% CI = [0.09, 0.24], *p* < 0.001) positively predicted adolescents’ depression level, whereas SMU intensity (β = –0.03, SE = 0.04, 95% CI = [–0.11, 0.06], *p* = 0.498) and active SMU (β = –0.06, SE = 0.04, 95% CI = [–0.14, 0.03], *p* = 0.162) did not significantly predict adolescents’ depression level.

**Table 2:** Fit indices for social media use profiles in adolescents

Number of profiles	AIC	BIC	aBIC	Entropy	LMR	BLRT	Smallest profile
1 Profile	23,965.997	24,014.934	23,983.174	–	–	–	100% (*N* = 844)
2 Profiles	22,995.631	23,073.930	23,023.113	0.713	959.176***	982.366***	47.77% (*n* = 471)
3 Profiles	22,632.617	22,740.277	22,670.405	0.772	366.162***	375.015***	20.69% (*n* = 204)
4 Profiles	22,564.144	22,701.166	22,612.238	0.715	78.573*	80.473***	8.11% (*n* = 80)
**5 Profiles**	**22,517.759**	**22,684.143**	**22,576.158**	**0.702**	57.007*	58.385***	**9.84% (*n* = 97)**
6 Profiles	22,483.255	22,679.001	22,551.960	0.696	45.406	46.504***	4.87% (*n* = 48)

The model in bold is the final model

*N* 986; *AIC * Akaike information criteria, *BIC* Bayesian information criterion, *aBIC* Adjusted BIC, *LMR* Lo-Mendell-Rubin adjusted likelihood ratio test, *BLRT* Bootstrapped likelihood ratio test; *p < .05, ***p < .001

### The Social Media Use Profiles Identified

The latent profile analysis was conducted to identify the profiles of social media use among adolescents. Table 2 shows the goodness-of-fit indices for the profiles with different numbers from one to six. The model with six profiles was excluded due to relatively low entropy [63], non-significant LMR, and the smallest profile representing less than 5% of the total sample [64]. The goodness-of-fit indices for the model with five profiles were better. Specifically, AIC, BIC, and aBIC were relatively small, the entropy was higher than 0.7, LMR and BLRT were significant which indicating a better fit compared to the four-profile model, and the smallest profile was considered reasonable [64]. Therefore, the model with five profiles was selected as the final model.

**Fig. 1:**
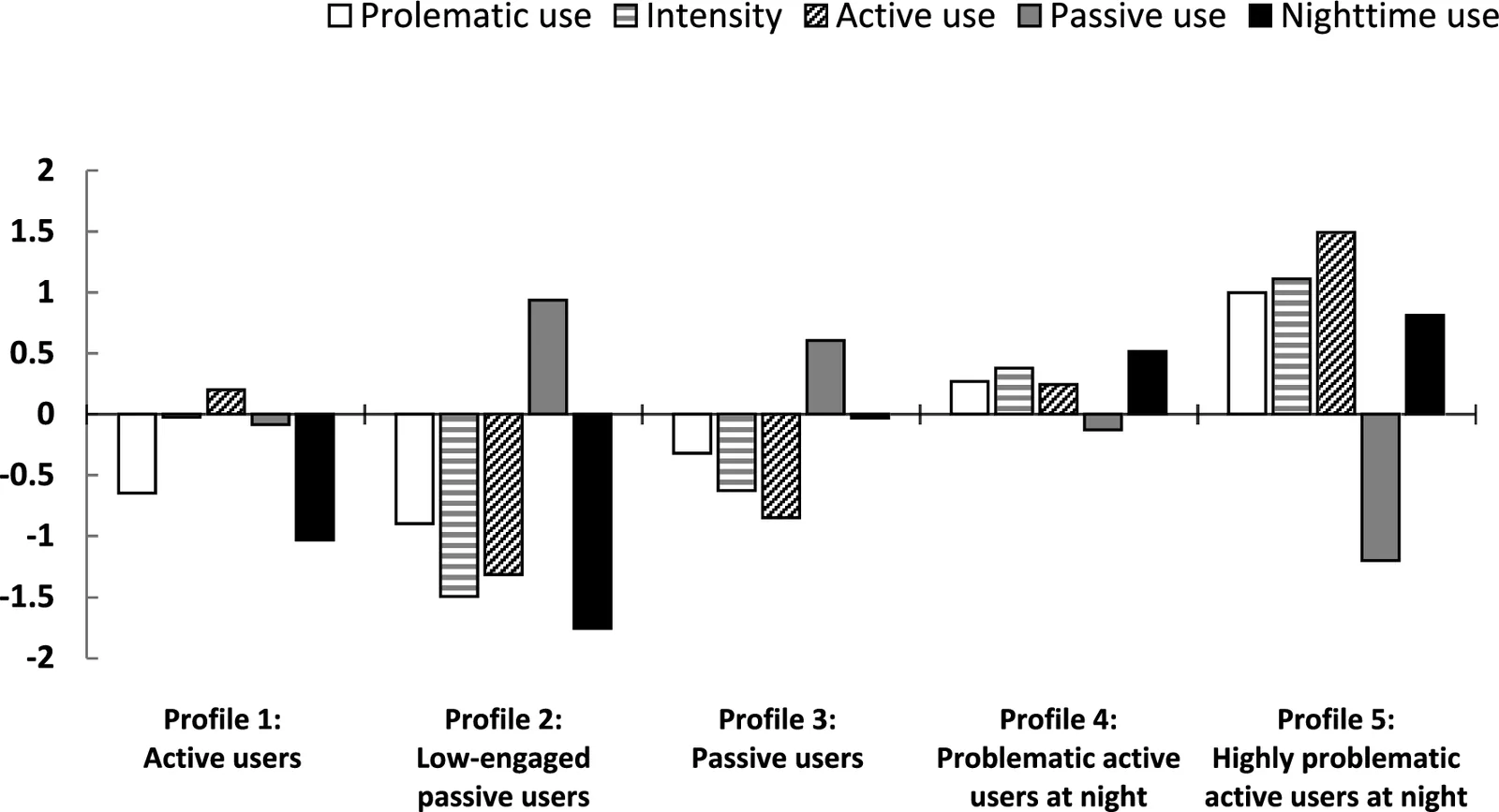
Z-scores of the indicators in each profile. *Note* The longitudinal axis is the z-score of each indicator

Then the differences of the profile indicators (i.e., problematic SMU, SMU intensity, active SMU, passive SMU, and nighttime SMU) were examined by MANOVA. As shown in Table 3, the adolescents in Profile 1 (*n* = 126, 12.8%) showed a relatively high level of active SMU, an average level of SMU intensity and passive SMU, and a relatively low level of problematic SMU and nighttime SMU. In Profile 2 (*n* = 97, 9.8%), the adolescents had the highest level of passive SMU, whereas the lowest level of problematic SMU, SMU intensity, active SMU, and nighttime SMU. In Profile 3 (*n* = 251, 25.5%), the adolescents used social media passively in a relatively high extent, and they had an average or moderate level of nighttime SMU and problematic SMU, and a relatively low level of SMU intensity and active SMU. In Profile 4 (*n* = 358, 36.3%), the adolescents' scores for problematic SMU, SMU intensity, active SMU, and nighttime SMU were relatively high, whereas the level of passive SMU was relatively low. The adolescents in Profile 5 (*n* = 154, 15.6%) were similar to those in Profile 4 but demonstrated significantly higher levels in each indicator. Based on this, these five profiles (i.e., Profile 1 to Profile 5 as illustrated in Fig. 1) were respectively labelled as *Active users*, *Low-engaged passive users*, *Passive users*, *Problematic active users at night*, and *Highly problematic active users at night.*

**Table 3:** Mean differences of the indicators in five social media use profiles

Indicators	*M* (*SD*)						*F*
	Total sample (*N* = 986)	Profile 1: Active users (*n* = 126)	Profile 2: Low-intensity passive users (*n* = 97)	Profile 3: Passive users (*n* = 251)	Profile 4: Problematic active users at night (*n* = 358)	Profile 5: Highly problematic active users at night (*n* = 154)	
SMU addiction	13.06 (4.33)	10.25^a^ (2.90)	9.16^b^ (3.02)	11.67^c^ (3.30)	14.22^d^ (3.70)	17.37^e^ (4.15)	126.80***
SMU intensity	4.47 (1.30)	4.44^a^ (0.93)	2.53^b^ (0.82)	3.66^c^ (0.79)	4.96^d^ (0.91)	5.91^e^ (0.80)	318.00***
Active SMU	3.39 (1.26)	3.64^a^ (0.68)	1.73^b^ (0.63)	2.32^c^ (0.57)	3.70^a^ (0.66)	5.27^d^ (0.77)	658.70***
Passive SMU	16.18 (3.86)	15.85^a^ (3.08)	19.79^b^ (3.04)	18.51^c^ (2.78)	15.68^a^ (2.97)	11.55^d^ (3.10)	171.54***
Nighttime SMU	14.75 (5.72)	8.83^a^ (2.82)	4.69^b^ (3.10)	14.56^c^ (3.59)	17.69^d^ (3.39)	19.40^e^ (3.92)	437.35***

The means with different superscripts different significantly with each other by profiles; ***p < .001

**Table 4:** Partially standardized relative indirect effects and 95% confidence intervals

Model path	Estimated effect	95% CI	
		Lower	Upper
P2 → SE → Depression	0.0694	− 0.0593	0.2012
P2 → SCC → Depression	− 0.0083	− 0.0578	0.0402
P3 → SE → Depression	0.3204	0.2075	0.4347
P3 → SCC → Depression	0.0887	0.0428	0.1435
P4 → SE → Depression	0.2613	0.1558	0.3645
P4 → SCC → Depression	0.1108	0.0622	0.1673
P5 → SE → Depression	0.1669	0.0431	0.2877
P5 → SCC → Depression	0.1159	0.0608	0.1803

P2 = Low-engaged passive users profile, P3 = Passive users profile, P4 = Problematic active users at night profile, and P5 = Highly problematic active users at night profile;  *SE*Self-esteem; *SCC * Self-concept clarity; 95% CI = 95% confidence intervals; Bootstrap sample size = 5000

### The Relative Mediating Effects From the Person-Centered Perspective

**Fig. 2:**
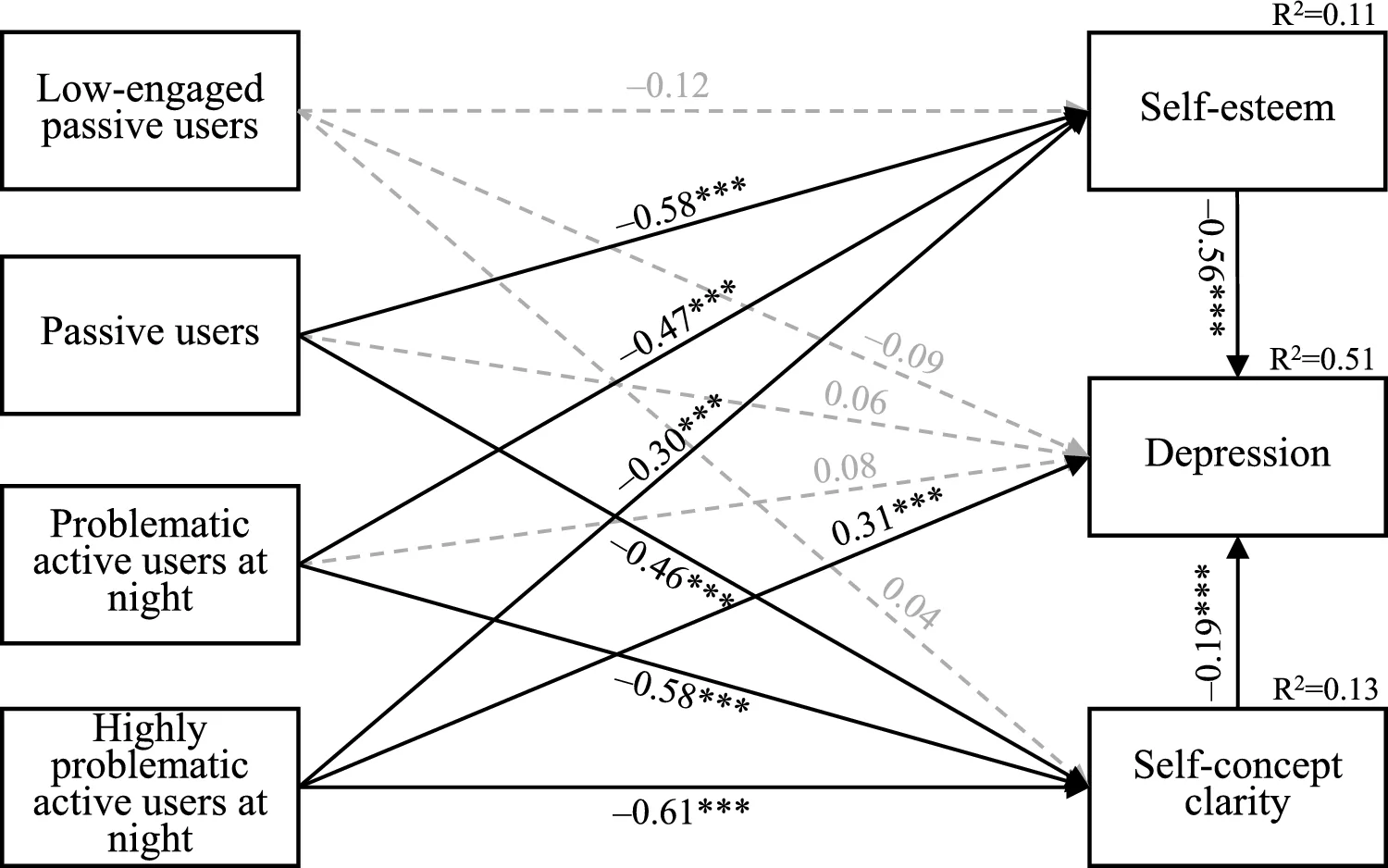
The relative mediating effects of self-esteem and self-concept clarity between social media use profiles and depression. *Note* The Active users profile was taken as the reference group. The path coefficients were standardized. Age and gender were controlled, whereas they were not shown in the figure to keep it concise

Then the *Active users* profile was taken as the reference group to analyse the relative indirect effects of self-esteem and self-concept clarity in the relationships between SMU profiles and depression. The results were depicted in Fig. 2. Compared to the adolescents in the *Active users* profile, adolescents in the *Passive users* profile, the *Problematic active users at night* profile, and the *Highly Problematic active users at night* profile tended to exhibit lower levels of both self-esteem and self-concept clarity, whereas no significant differences were found between the *Active users* profile and the *Low-engaged passive users* profile. Further, both self-esteem and self-concept clarity were negatively associated with depression. In addition, as shown in Table 4 the value of zero was not included in the 95% CIs of all model paths except for the paths starting from the *Low-engaged passive users* profile. Therefore, compared to the adolescents in the *Active users* profile, self-esteem and self-concept clarity played the significant relative mediators in the relationship between the SMU profiles (including *Passive users* profile, *Problematic active users at night* profile, and *Highly problematic active users at night* profile) and adolescent depression.

## Discussion

Based on theoretical (e.g., Beck’s cognitive theory of depression; [41, 42]) and empirical evidence, the present study examined the association between different types of adolescents’ social media use (i.e., problematic SMU, SMU intensity, active SMU, passive SMU, and nighttime SMU) and depression, as well as the mediating role of self-esteem and self-concept clarity in these associations using the person-centered approach. To our knowledge, this study stands as a novel research endeavor to concurrently examine these five types of SMU and their effects on adolescent depression. The present study yielded several noteworthy findings, which are elaborated upon in the following discussion.

Firstly, this study revealed that various forms of SMU played an important role in adolescent depression. Specifically, problematic SMU, passive SMU, and nighttime SMU were found to be directly and positively related to adolescents’ depression. This finding is in line with previous studies (e.g., [20, 26, 67, 68]). Problematic/addiction-like ways of SMU [67, 68], passive ways of SMU [12, 28], and SMU during the nighttime [25, 26] are correlated with more psychosocial problems. These relationships remained significant even after controlling for other forms of SMU, supporting the robustness and uniqueness of the similar findings in previous studies. However, we did not find a significant association between the intensity of SMU and depression, nor between active SMU and depression. One possible interpretation for these non-significant results is that SMU intensity and active SMU may act as both protective and risk factors in the development of depression among adolescents. For SMU intensity, its higher level predicted more problematic ways of SMU one year later [67] which is a risk factor for depression, whereas higher frequency or intensity of SMU is also related to more social interaction and social capital/support [67, 69, 70] potentially buffering its harmful effects. For active ways of SMU (e.g., posting, messaging), it enhances the sense of connectedness and increases social capital [71] which may reduce the likelihood of developing depression. However, the lack of positive feedback following active SMU could potentially increase depression levels, given that positive feedback is an essential prerequisite for adolescents to benefit from such engagement [58]. The intertwined protective and risk effects appear to have conflated in the pathways from both SMU intensity and active SMU to depression, rendering them nonsignificant in this study. Subsequent research endeavors should aim to disentangle and further elucidate these complex relationships.

Secondly, in our sample of Italian adolescents, five distinct profiles of social media use were identified: (1) the *Active users* profile (*n* = 126, 12.8%), (2) the *Low-intensity passive users* profile (*n* = 97, 9.8%), (3) the *Passive users* profile (*n* = 251, 25.5%), (4) the *Problematic active users at night* (*n* = 358, 36.3%), (5) the *Highly problematic active users at night* profile (*n* = 154, 15.6%). A direct comparison of our findings on SMU profiles with those in previous similar studies is difficult due to differences in sample characteristics and profile indicators, however, there are some noteworthy points in our results that need to be highlighted. For example, adolescents in Profile 1 displayed an average level of SMU intensity, which was significantly higher than that observed in Profile 3, whereas they had a lower level of problematic SMU compared to the adolescents in Profile 3. This result reveals that a high intensity of SMU is not necessarily a prerequisite for a high and problematic dependence on social media. This is consistent with the perspective emphasized in previous similar studies, stating that the problematic/addictive ways of SMU and the intensity of SMU should be considered as distinct dimensions [67]. Another noteworthy finding is the lack of a significant difference in depression levels between Profile 4 and Profile 5. Although the levels of problematic SMU and nighttime SMU which positively predicted depression were higher in Profile 5 than in Profile 4, the passive SMU level which is also a positive predictor of depression was much lower in Profile 5 than in Profile 4. This finding indicated that when investigating the impact of SMU on adolescents, it is crucial to consider multiple indicators, rather than relying on single indicator, to truly uncover the complex relationships. This approach may also contribute to addressing the issue of heterogeneity in conclusions observed in previous studies [17].

Finally, the relative mediating roles of self-esteem and self-concept clarity were discovered. Taking the *Active users* profile as the reference group, self-esteem and self-concept clarity mediated the relationships between the SMU profiles (i.e., the *Passive users* profile, the *Problematic active users at night* profile, and the *Highly problematic active users at night* profile) and depression. This is in line with the self-concept fragmentation hypothesis [33, 34] and Beck’s cognitive theory of depression [41, 42]. In the first phase of the mediation process, one noteworthy result is that adolescents in the *Low-engaged passive users* profile, who exhibited higher levels of passive SMU, did not have lower levels of self-esteem and self-concept clarity compared to the adolescents in the *Active users* profile. This may due to the relatively low levels of problematic SMU, SMU intensity, active SMU, and nighttime SMU in the *Low-engaged passive users* profile. In the second stage of the mediating process, adolescent depression was negatively predicted by both self-esteem and self-concept clarity. This finding is consistent with previous research (e.g., [44–46]). Adolescents with lower self-esteem and lower self-concept clarity may exhibit higher vulnerability in adapting to negative affect and stress [72], which ultimately leads to an increase in depressive symptoms [41, 42].

There are several limitations in this study should be noted. First, social media platforms play a role in the relationship between SMU and depression [13], but we only focus on general social media platforms. Future studies can further explore the use patterns of specific social media platforms and their effects. Second, due to the cross-sectional nature of the present study, no definitive conclusions can be drawn regarding the directionality and causality of the relationships between the studied variables. Future research endeavours should incorporate longitudinal and/or experimental methods to further elucidate these relationships. Third, following the extended active-passive model of SMU [71], active SMU and passive SMU can be further decomposed. Future studies could focus on more specific aspects of active SMU and passive SMU, as well as their associated effects. Fourth, during data collection, the total number of schools approached was not recorded in this study, making it impossible to calculate the response rate at the school level. Future studies should track this information. Finally, this study included only adolescents from schools located in northern Italy, which limits the generalizability of our findings to adolescents from central or southern Italy or other countries/cultures. Nationwide or cross-cultural studies are recommended to systematically explore potential cultural differences in these aspects.

## Data Availability

The datasets generated and/or analysed during the current study are available on reasonable request.
